# The Biodegradation of Acrylic-Coated Woven Fabrics by *Gordonia alkanivorans S7*: A Novel Approach for Sustainable Textile Waste Management

**DOI:** 10.3390/ma18081745

**Published:** 2025-04-10

**Authors:** Marcin Henryk Struszczyk, Magdalena Olejnik, Agnieszka Gutowska, Edyta Chmal-Fudali, Olga Marchut-Mikołajczyk, Katarzyna Struszczyk-Świta, Piotr Drożdżyński

**Affiliations:** 1 Institute of Security Technologies “MORATEX”, 3 M. Sklodowskiej-Curie Str., 90-505 Lodz, Poland; mstruszczyk@moratex.eu (M.H.S.); molejnik@moratex.eu (M.O.); efudali@moratex.eu (E.C.-F.); 2 Institute of Molecular and Industrial Biotechnology, Faculty of Biotechnology and Food Sciences, Lodz University of Technology, 2/22 Stefanowskiego Str., 92-537 Lodz, Poland; olga.marchut-mikolajczyk@p.lodz.pl (O.M.-M.); katarzyna.struszczyk@p.lodz.pl (K.S.-Ś.); piotr.drozdzynski@p.lodz.pl (P.D.)

**Keywords:** microbial degradation, coated woven fabric, textile waste, *Gordonia alkanivorans S7*

## Abstract

The increasing environmental issue related to textile waste, especially synthetic fibers treated with acrylic resins, demands the creation of sustainable recycling techniques. Biotechnological methods, such as microbial degradation, present a viable solution for the elimination of these coatings and the recovery of important fibers. This study investigates the potential of a biotechnological approach for the removal of acrylic resins from coated woven textile wastes. The biodegradation process of coated woven fabric after the pretreatment at a high temperature (121 °C) or 6% H_2_O_2_ was performed using the hydrocarbon-degrading bacterial strain *Gordonia alkanivorans S7*. Over a 72 h biodegradation period, an increase in emulsifying and esterase activities was observed. A reduction mass of the coated textile waste by up to 7 wt% was achieved, and the elimination of acrylic resin was confirmed through FTIR analysis. The findings indicate the usefulness of the biotechnological method in eliminating acrylic resin from textile waste, presenting a viable strategy for polyester fiber recovery and substantially mitigating its environmental impact.

## 1. Introduction

Rising energy and water consumption and the resulting environmental pollution are major challenges of the twenty-first century [1]. In 2020, the textile industry ranked as the third highest contributor to water and land degradation. In that year, each EU resident required an average of nine cubic meters of water, 400 square meters of land, and 391 kg of raw materials to produce clothing and footwear. In addition, during the entire production process, various types of chemicals, which are often dangerous for the environment, are used and released, and the resulting pollutants contribute to the deepening climate change [2,3,4]. The European Environment Agency reports that textile purchases in the EU in 2020 resulted in around 270 kg of CO_2_ emissions per capita. Textile products consumed in the EU produced a total of 121 million tons of greenhouse gas emissions.

An important problem is also the way of managing textile and clothing waste, because the rapid increase in the consumption of these types of products (especially clothing) and thus the waste resulting from their use caused the need to increase the area of landfills, as well as intensify their disposal, mainly through incineration [2,5,6,7]. Textile manufacturing is estimated to account for around 20% of global freshwater contamination due to dyeing and finishing processes [2].

Many scientific studies address the topic of textile preservation, mainly in the aspect of chemical pollution of discharged waste water [8], pollution of sewage and groundwater, and consequently, large water tanks with microplastics/microfibers released during maintenance [2,3,9,10,11,12,13]. These issues negatively impact aquatic habitats and the associated fauna and flora.

In 2015, the European Parliament approved Directive 2018/851, which amended Directive 2008/98/EC, according to which, apart from previous provisions, from 2025, all EU countries are obliged to segregate textile waste [2,14]. In addition, producers are fully responsible for managing the waste generated by recycling or disposing it [14].

Therefore, the literature increasingly focuses on the recycling of textile materials [7,8,13,14,15,16,17], particularly highlighting pro-ecological biotechnological approaches [3,5,9,18].

To improve the durability of polyester products, synthetic compounds like polyacrylic resins are commonly used to coat fabrics to improve properties such as color fastness and resistance to environmental factors. However, the widespread use of these coated fabrics generates substantial post-production waste. The presence of synthetic coatings renders the waste resistant to degradation and inhibits material recycling. Removing polyacrylic resin from textile surfaces could facilitate material reuse, thereby reducing waste and offering significant ecological benefits. To date, the degradation of acrylic resins has mainly been carried out using chemical or thermal methods [19,20,21]. These methods are burdensome for the natural environment and generate toxic waste. In addition, chemical and thermal methods require the use of high temperatures, which significantly increases the cost of the process and the possibility of damaging the structure of the material and losing their basic, desired properties. One of the key chemical factors influencing acrylic resins is hydrogen peroxide (H_2_O_2_). De Oliveira et al. (2010) explored its potential application as a disinfectant in dentistry and evaluated its effects on acrylic resins. Their findings indicate that exposure to hydrogen peroxide can lead to adverse effects, including color changes and a reduction in flexural strength [22,23]. This degradation of the polymer matrix increases surface roughness, thereby facilitating microbial colonization [24].

Biotechnological processes, including biodegradation, serve as alternatives to chemical methods. Biodegradation refers to the disintegration of organic chemicals into simple inorganic substances, ultimately resulting in carbon dioxide and water, and is facilitated by microorganisms [25,26,27]. The literature primarily explores the biodegradation of acrylic resins through the perspective of biodeterioration, emphasizing the detrimental effects of microorganisms on these materials in dentistry [28,29] and their environmental degradation [28,29,30]. In this context, enzymatically catalyzed reactions alter the chemical and physical properties of acrylic polymers, influencing both their structural integrity in biomedical applications and their breakdown in ecological systems. In the case of the biodegradation of acrylic polymers, as a result of the reactions catalyzed by enzymes, the chemical and physical properties of the polymer change. At the initial stage of the process, low-molecular-weight compounds released from the polymer structure are transported into the cells of the microorganisms conducting the process, as they are a source of carbon and energy [31]. The biodegradability of acrylic polymers depends on their structure, environmental conditions, the type of microorganism, and its degradation activity relative to the polymer [31,32,33,34]. Moreover, the microbial resistance index, which evaluates the proliferation of microorganisms on a scale of 0 to 3, with 3 indicating robust growth, is 0.51 for acrylic resins, signifying their resistance to microbial degradation [34]. The bioavailability of acrylic polymers may be enhanced by combining microbial degradation with chemical pretreatment. It was proven that the application of hydrogen peroxide in the Fenton process markedly enhances the biodegradability of waste generated from acrylic fiber manufacture [35].

Biotechnological methods have not yet been applied to remove polyacrylic resins from polyester textile surfaces, a significant waste material. The biodegradation of acrylic resins from textiles is more complex than the degradation of the polymers alone, as it must account for both the resin structure and the underlying material. A method that minimizes the impact on fabric properties while effectively removing the acrylic polymer needs to be developed. Bioformulations for the removal of resin layers from fabrics represent a viable alternative to chemical methods. Biotechnological methods are environmentally friendly as the microorganisms utilized are sourced from the natural environment. The soluble degradation products of polymers associated with waste materials represent a natural method for their utilization in the environment. The end products of this typically prolonged process are biomass, carbon dioxide, and water. The biotechnological method avoids the introduction of new chemical impurities into the environment, such as solvents and synthetic surfactants, in relation to the substances present in the production waste. The objective of this research was to develop a laboratory-scale biodegradation process for coated woven fabrics using the *Gordonia alkanivorans S7* bacterial strain to remove acrylic resins from the surface of textile products. In addition, the study aimed to compare the results of the biodegradation process with those achieved by a chemical method of resin removal from the coated surface.

## 2. Materials and Methods

### 2.1. Materials

#### 2.1.1. Textile Material

The research focused on polyester woven fabrics coated on one side with acrylic resin, categorized as post-production/post-use waste from Miranda S.A. in Turek, Poland. The materials consisted of waste from umbrella sheathing waste as well as cuttings, slices, and base fabrics. Due to the fact that the research was of an application character, samples of waste-coated textiles were provided from commercial entities without information about the actual chemical composition due to trade secrets.

#### 2.1.2. Biological Material

The biological material utilized in the investigation was a bacterial strain of *Gordonia* from the resources of the Institute of Molecular and Industrial Biotechnology at the Lodz University of Technology. This bacterial strain was isolated from petrochemical wastewater and is characterized by high degradation activity relative to a wide spectrum of hydrocarbon compounds [36,37].

### 2.2. Methods

#### 2.2.1. Biodegradation Experiments

Before the biodegradation process, samples of textile materials were cut into equal fragments and sterilized at 120 °C for 20 min (SYSTEC VE-75, Systec GmbH & Co. KG, Linden, Germany). The biodegradation process was carried out in MSM mineral medium-submerged culture conditions.

Forty cm^3^ of liquid MSM medium, free of a carbon source, was dispensed into 500 cm^3^ flasks and sterilized at 121 °C for 17 min. Prior to sterilization, the substrate’s pH was adjusted to 6.5. The removal of resin from the surface of the material was conducted in four variations: SB—the material was subjected to sterilization (17 min at 121 °C) and then biodegraded utilizing the tested bacterial strain. SHPB—the material was subjected to sterilization (17 min at 121 °C), subjected to a 30 min treatment with 6% H_2_O_2_, subsequently rinsed with sterile distilled water, and then biodegraded. SHP—the material was subjected to sterilization (17 min at 121 °C) and thereafter treated for only 30 min in 6% H_2_O_2_; S—the material was subjected to sterilization only (17 min at 121 °C). The control sample (C sample) was the original sample of coated woven fabric.

Samples of the material were placed in flasks containing a liquid medium under sterile conditions, followed by the inoculation of *Gordonia alkanivorans S7* in variants 1 and 2. The inoculum was derived from a 24 h plunge culture of the S7 strain. The inoculum was utilized at a volume of 0.6 cm^3^ (OD600 approximately 1.4) per flask. In option 3, the medium remained uninoculated. The flasks were placed on a shaker where the biodegradation process took place at a temperature of 30 °C and a speed of 200 rpm. After three days, the biodegradation process was complete. The control sample consisted of sterilized material fragments from flasks that were placed in the medium without the addition of inoculum.

The biodegradation process was monitored by measuring pH changes, analyzing microbial growth by protein concentration using Bradford’s method [38], evaluating the emulsifying activity [39], and examining changes in esterase activity in the culture medium [40].

#### 2.2.2. Mass Reduction

The mass of the fabric samples was determined before and after the coating removal process using a biotechnological method, following the PN-EN ISO 2286-2:2016-11 standard guidelines [41]. 

Five samples measuring 10 × 10 cm were extracted from the uncoated material under examination, and the surface area of each sample was computed with an accuracy of 1 mm^2^.

Each test sample was weighed to the accuracy of 0.01 g. The total surface mass (*m_p_*), expressed in [g/m^2^], is calculated using the following formula:

(1) $$m_{p}=\frac{m\cdot 10^{6}}{A}$$

where

*m_p_* is the areal density of the uncoated textile material [g/m^2^];

*m* is the mass of the tested sample [g];

*A* is the areal density of the working sample expressed in [mm^2^].

Five samples of 10 × 10 cm in size were cut from the coated material under test; then, the surface area of each working sample was calculated with an accuracy of 1 mm^2^. Each test sample was weighed to the nearest 0.01 g. The total surface mass (m_pp_), expressed in g/m^2^, is determined according to the following formula:

(2) $$m_{pp}=\frac{m\cdot 10^{6}}{A}$$

where

*m_pp_* is the areal density of the coated textile material [g/m^2^];

*m* is the mass of the tested sample [g];

*A* is the areal density of the working sample expressed in [mm^2^].

Finally, the average of the five samples determined was calculated, expressing the final result with an accuracy of 1 g/m^2^. The total coating mass (*m_po_*), expressed in [g/m^2^], is calculated according to the formula:

(3) $$m_{po}=m_{pp}-m_{p}$$

where

m_pp_ is the areal density of the coated textile material [g/m^2^];

m_p_ is the areal density of the uncoated textile material [g/m^2^];

m_po_ is the coating mass [g/m^2^].

#### 2.2.3. Construction Characteristics of Coated Woven Fabric Before and After the Biodegradation Process: An ATR-FTIR Study

ATR-FTIR tests were performed using the FTIR–Nicolet IS10 single-beam spectrophotometer (Thermo Fisher Scientific, Waltham, MA, USA) equipped with a computer set and the OMNIC 9.2.86 data acquisition software.

Due to the specificity of the samples tested, the attenuated total reflection (ATR) technique was used, applying an ATR attachment equipped with a Smart iIR-type reflective attachment and a diamond crystal with a reflection angle of 45°.

The experiment began by recording the background spectrum (air), which served as the reference FTIR spectrum. In order to obtain the most accurate results possible, the background spectrum was deducted from the spectrum obtained in the actual experiment. ATR-FTIR specks were recorded on the absorbance scale in the wavenumber range from 4000 cm^−1^ to 650 cm^−1^ with a resolution of 4 cm^−1^. For each test sample, 32 scans were performed to eliminate accidental interference.

The analysis of the recorded oscillatory rotation spacers allowed us to conclude about possible structural changes in the tested samples. The chemical structure was determined based on the analysis of absorption bands occurring in the spectra and identification schemes taken from the OMNIC 8.0.380 software.

## 3. Results

While acrylic resins are thought to be biodegradable and certain microbes have demonstrated the capacity to digest them, it is important to recognize that these polymers are extensively utilized, and their degradation byproducts could pose a threat to the environment. The biodegradation process is affected by various physicochemical and biochemical variables [33].

### 3.1. pH Analysis

**Figure 1:**
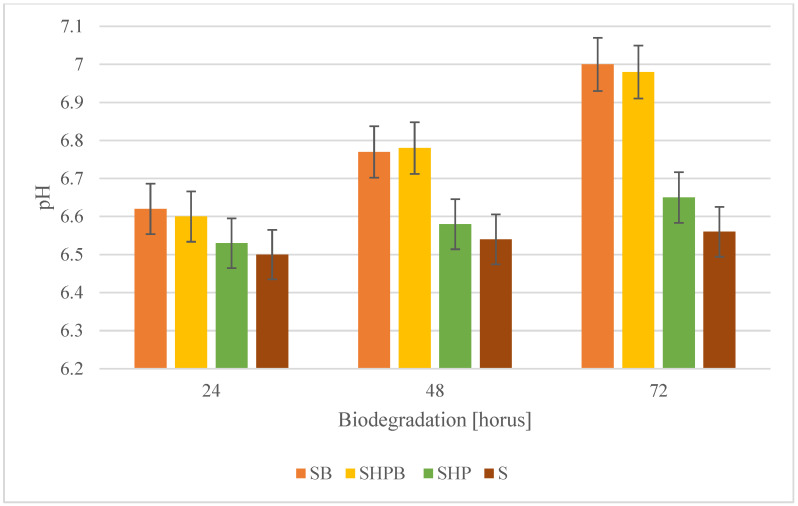
pH changes in the culture medium during 72 h of biodegradation of coated woven fabrics. SB—the material was sterilized and then biodegraded using the tested strain of bacteria; SHPB—the material was sterilized, treated for 30 min in 6% H_2_O_2_, washed with sterile distilled water, and then biodegraded; SHP—the material was sterilized and then treated only for 30 min in 6% H_2_O_2_; S—the material was sterilized. The mass reduction for samples 3 and 4 was not observed in all cases of the treatment.

The pH level is a critical experimental parameter influencing biodegradation efficiency, significantly impacting bacterial nutrition consumption, interactions with organic contaminants, and enzyme production capacity [42]. Figure 1 illustrates the changes in the pH of the medium during the biodegradation process of acrylic resins on woven textile items, conducted by the strain *Gordonia alkanivorans S7* using various material treatments. Changes in the pH value of the culture medium may validate the biodegradation process. Notable alterations in pH were detected in culture media including samples of coated woven fabric that underwent heat treatment (sterilization) prior to the biodegradation procedure. An increase in pH during the process may signify the appearance of breakdown products from resins as well as biosurfactants in the environment. Markedly diminished alterations in the pH of the culture media were seen for samples treated with 6% hydrogen peroxide. This phenomena may result from the improper selection of the chemical treatment process, as evidenced by the absence of pH changes in uninoculated samples. No changes in the parameters studied were observed in the control trials.

The pH values during biodegradation seems to be aligned with literature data. According to Grabowska et al., 2012 [43], during acrylic polymer biodegradation, the pH should be at a level of 7–8. Also, slightly acidic/alkaline and neutral conditions were determined to be suitable for the degradation of acrylic compounds, which is likely associated with enzymes that facilitate efficient microbial degradation within a pH range of 6–9 [44].

### 3.2. Microbial Growth

To evaluate the potential adhesion of bacterial biomass to the surface of coated woven fabric, an indirect method was employed to assess microbial growth through the analysis of protein concentration in the culture media using Bradford’s method [38].

The results of protein determination in the culture media are presented in Table 1.

A growth rate of nearly 1.5 times higher for the *G. alkanivorans S7* bacteria strain was recorded in trials where the biodegradation process of coated woven fabric, which underwent only sterilization, was conducted. The application of the chemical treatment utilizing 6% hydrogen peroxide led to a decrease in the growth rate of the microorganism involved in the process. This is likely associated with the residual hydrogen peroxide present on the material and its detrimental impact on microorganisms. Hydrogen peroxide (H_2_O_2_), even at a low concentration of 3%, exhibits a high degree of stability at a neutral pH and ambient temperature. It generates highly reactive hydroxyl radicals capable of eliminating any microbial cell. Low protein concentration values in samples following physical or physicochemical treatment suggest the release of protein substances from the test [45].

### 3.3. Emulsifying Activity and Esterase Activity Changes

The hydrophobic character of acrylic resins influences their bioavailability. Acrylic resins covering textile materials belong to hydrophobic compounds, which makes them difficult to access for microbial cells that carry out the biodegradation process. In the case of this type of compound, the biodegradation process may be supported by surface-active compounds (biosurfactants) produced by microorganisms [46]. In order to investigate the ability of the *G. alkanivorans* S7 strain to produce biosurfactants during the biodegradation process of coated textile materials, the emulsifying activity was investigated [39]. The highest values of emulsifying activity were obtained for biodegradation samples without the pretreatment with hydrogen peroxide. However, after 72 h of the process, the values of emulsifying activity obtained for non-pretreated and pretreated samples were similar, with an OD_500_ of 0.62 and an OD_500_ of 0.58, respectively. The trace values of the emulsifying activity obtained for the control samples are most likely related to the mechanical influence on the material during the shaking process and the release of waste components. Salman et al. [47] investigated the influence of biosurfactants on the mechanical properties of acrylic resins. Their findings indicated that the hardness of acrylic diminishes due to the influence of biosurfactants. The interaction of the oxidizing agent with the biosurfactant diminishes the strength of the polymer chains. However, the effect of the oxidizing agent alone does not influence the softening of acrylic resin [48].

**Figure 2:**
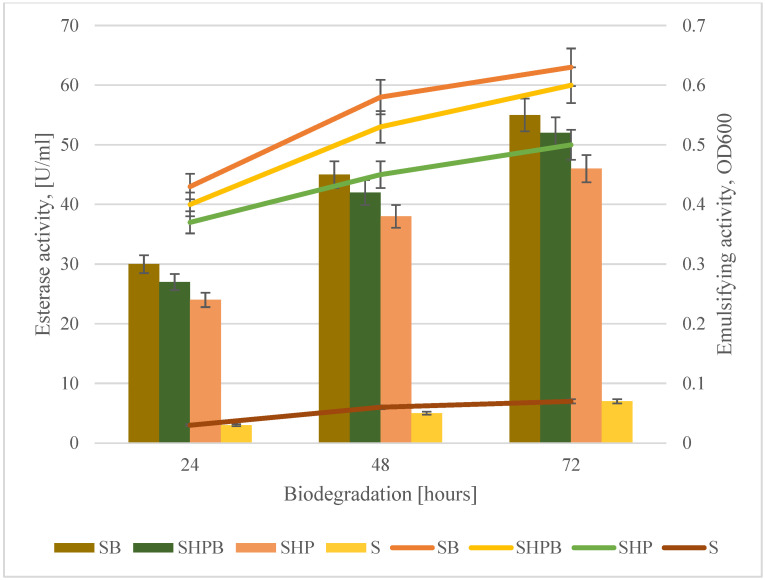
Changes in emulsifying activity and esterase activity during 72 h of biodegradation of coated woven fabrics. SB—the material was sterilized and then biodegraded using the tested strain of bacteria; SHPB—the material was sterilized, treated for 30 min in 6% H_2_O_2_, washed with sterile distilled water, and then biodegraded; SHP—the material was sterilized and then treated only for 30 min in 6% H_2_O_2_; S—the material was sterilized. The mass reduction for samples 3 and 4 was not observed in all cases of the treatment.

During the 72 h biodegradation process, an increase in esterase activity both for nonpretreated and pretreated samples was noted (Figure 2). Several authors point to the important role of esterase in the biodegradation of acrylic polymers [33,49,50]. Genes encoding esterases and amidases have been found in microorganisms exhibiting the biodegradability of acrylic composites, suggesting that these proteins are involved in the degradation of these polymers. Guo et al. investigated the role of esterases on the biodegradation of acrylic resins used in dental composites [47]. Bacterial esterases affect the mechanical properties of the resin, reducing the tensile strength and elasticity of the resin composite, its microhardness, flexural strength, radial tensile strength, and modulus of elasticity. When the resin surface becomes abrasive, the rate of biodegradation increases due to increased bacterial adhesion [51,52]. Although the mechanisms are not fully understood, it is believed that these enzymes attack the side chains of acrylic polymers, resulting in degradation intermediates which are then transferred to the oxidation pathway [33,53].

In the trials for which the highest esterase activity was observed, the highest values of emulsifying activity were also found. This phenomenon may be related to the formation of intermediates in the biochemical decomposition of acrylic resins, which themselves can act as emulsifiers. Furthermore, Kumagai et al. (2018) observed that esterase alone can be an optimal emulsifier [54]. Increased emulsifying activity in the culture fluid may be related to the production of surfactants by the *Gordonia alkanivorans S7* strain [36,37].

### 3.4. Mass Reduction

The resin coating on the fabric is a crucial component of the waste and influences its weight. To verify the breakdown of the acrylic resin layer on the surface of the material, the samples were weighed prior to and subsequent to the chemical treatment, as well as before and after the biodegradation process. After 24 h of the biodegradation process of the coated woven fabric, in trials inoculated with the *Gordonia alkanivorans S7* bacterial strain, without the pretreatment with hydrogen peroxide, the appearance of lint in the substrate was observed. This was probably part of the resin removed from the surface of the material. After the 72 h biodegradation process, in the trials in which the material samples were sterilized and then biodegraded using the tested strain, a loss of sample weight was observed, while no loss was observed for the SHP and S samples. In the case of the SHPB sample, which was additionally treated with 6% H_2_O_2,_ a loss of mass of the material samples and macroscopic changes in the substrate were observed.

The mass reduction in the coated woven fabric treated with different methods is presented in Table 2.

The highest mass reduction of 6.90 wt% was noted for samples treated with the *Gordonia alkanovorans S7* bacteria strain.

The pretreatment with 6% H_2_O_2_ before bacterial inoculation did not significantly increase the reduction in sample mass during biodegradation. A mass loss of 5.81% by weight was observed in these experiments. Although the oxidizing agent may influence the loosening of the polymer structure, residual hydrogen peroxide was reported to have a detrimental effect on *G. alkanivorans* S7, leading to a slight decrease in process efficiency. The effect of H_2_O_2_ on the mass reduction in polyacrylic acid (PAA) was reported by Miyazaki et al. (2018). They observed the effect of a 10 mM hydrogen peroxide solution on the reduction in the molecular weight of PAA during 10–90 min of incubation [55].

Bankeeree et al. (2021) investigated the biodegradation of polyacrylamide pretreated with hydrogen peroxide in soil. H_2_O_2_ was used at concentrations of 0%, 4%, 8%, and 12% (*v*/*w*). The highest degree of degradation of polyacrylamide, which resulted in the highest waste loss, was observed for 12% (*v*/*w*) of hydrogen peroxide [56].

In our studied cases (the SHP and S samples), the absence of mass reduction was observed, but probably an increase in the roughness of the material surface occurred. This has a positive effect during the biodegradation process, because it facilitates bacterial colonization. 

### 3.5. ATR-FTIR Study

FTIR analyses were performed to investigated the impact of two initial factors—sterilization and hydrogen peroxide treatment—on the biodegradation process of coated textiles, with a specific focus on the removal of the acrylic coating. The application of these factors aimed to enhance the efficiency of the biodegradation process by facilitating the reduction in the fabric coating. The ATR-FTIR spectra of the coated woven fabric show the absorption bands characteristic of PES (Table 3) with one difference: the appearance of strong absorption bands at a wavenumber of 1158 cm^−1^, which corresponds to the C-O bond. For the analysis, the spectra of samples SB and SHPB were taken due to the detected above-mentioned spectra differences with the initial (C—control sample) sample of the coated woven fabric.

The ATR-FTIR spectrum of initial coated woven fabrics (C) was characterized by a wide absorption band at 3350 cm^−1^, which corresponded to the hydroxyl group that originated from the carboxy group, with two absorption bands at 2957 cm^−1^ and at 2931 cm^−1^ related to the -CH_2_ group. The C=O (related to the carboxyl group of ester) bond was detected at 1726 cm^−1^, whereas C=C originating from the benzene ring at 1579 cm^−1^ and 722 cm^−1^ was found, confirming the presence of an absorption band at 1379 cm^−1^ (aromatic ring). Moreover, the absorption bands at 1241 cm^−1^ and at 1158 cm^−1^ are related to C-O bonds, while the presence of C-O-C is confirmed by the absorptions at 1098 cm^−1^ and C-OH at 1018 cm^−1^.

The FTIR spectrum of polyester shows the absorption bands corresponding to alcohol, ester, anhydride (1713 cm^−1^; 1017 cm^−1^; 1240 cm^−1^;), and the aromatic ring (1408 cm^−1^) [57,58].

The above-mentioned indication confirms that the process of the biodegradation with or without hydrogen peroxide pretreatment resulted in the elimination of the polyacrylic coatings.

The presence of the layer of acrylic coating is confirmed by the absorption band at 1158 cm^−1^, which corresponds to the C-O bond, as well as by the strong absorption band at 2930 cm^−1^ (C-H stretching vibration) [59,60]. The absence of the bands at approx. 1155 cm^−1^ was detected in the FTIR spectra of the SB and SHPB samples subjected to biodegradation. The other characteristic absorptions for acrylic resin (such as the C=O and C-O bonds detectable at approx. 1720 cm^−1^, C=C at approx. 1637 cm^−1^, and C-C at approx. 1434 cm^−1^) are overlapped by the peaks of the polyester fibers.

**Figure 3:**
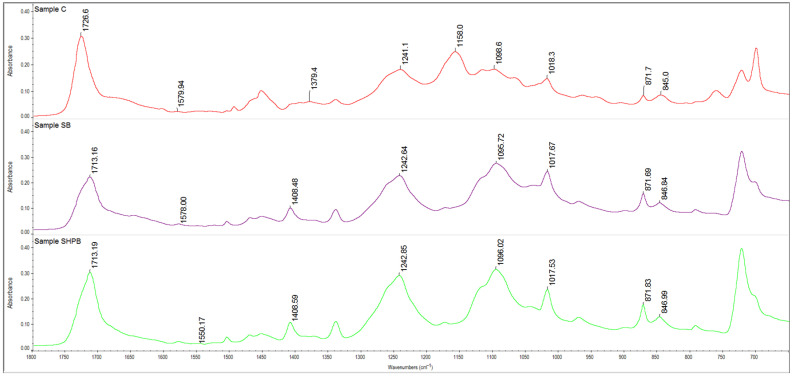
The ATR-FTIR spectra of the samples in the range of 2000 cm^−1^–650 cm^−1^: C—the control sample was made of initial coated woven fabric; SB—the coated textile fabric was sterilized and then biodegraded using the tested strain of bacteria; SHPB—the material was sterilized, treated for 30 min in 6% H_2_O_2_, washed with sterile distilled water, and then biodegraded.

The treatment of the *Gordonia alkanivorans S7* bacteria strain resulted in the disappearing of the absorption band at 1158 cm^−1^ and bands shifting related to the C=O carboxyl from the ester group to higher wavenumbers (appeared at 1713 cm^−1^) (Figure 3).

Moreover, the increase in the C=O carboxyl band resulting from the ester group, appearing for the initial coated textile at 1720 cm^−1^, to a higher wavenumber (1713 cm^−1^) is also the effect of the biodegradation process.

**Figure 4:**
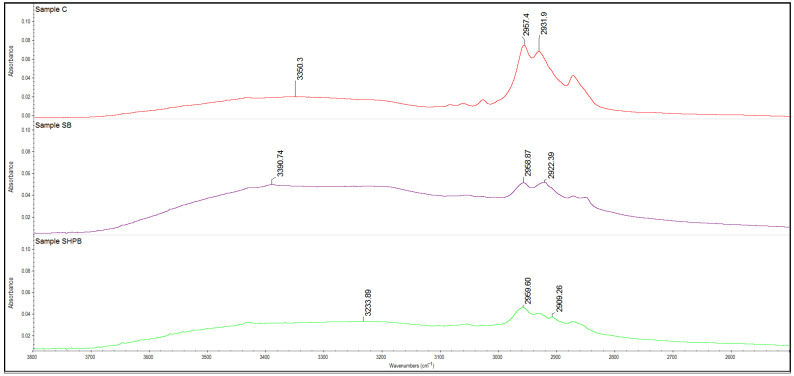
The ATR-FTIR spectra of the samples in the range of 3800 cm^−1^–250 cm^−1^: C—the control sample included initial coated woven fabric; SB—the coated textile fabric was sterilized and then biodegraded using the tested strain of bacteria; SHPB—the material was sterilized, treated for 30 min in 6% H_2_O_2_, washed with sterile distilled water, and then biodegraded.

The H_2_O_2_ pretreatment resulted in the shift of -OH absorption to the higher wavenumber (3233 cm^−1^), whereas biodegradation without the H_2_O_2_ treatment resulted in a reduction in the discussed absorption band to a lower wavenumber (3390 cm^−1^) (Figure 4).

An additional process of the H_2_O_2_ treatment combined with the biodegradation process did not caused any significant changes in the FTIR spectrum as compared with the sample treated only by the *Gordonia alkanivorans S7* strain bacteria. It can be summarized that the biodegradation process that preceded the H_2_O_2_ pretreatment resulted in a similar (but not identical in quantity) alteration in the ATR-FTIR spectrum, as observed for the biodegraded sample (confirmed by the loss of mass). Moreover, the application of the additional step of the H_2_O_2_ treatment prior to the biodegradation process did not affect the significant changes in the ATR-FTIR spectrum of the resulting textile.

The process of the degradation of the polyacrylic coating of the textiles depends significantly on the processing aids, as the additive enhances the functionality of the textiles [60]. The implementation of the above-mentioned substances (such as flame retardants) altered the behavior of the coating against the physical and biological degradation agents detectable during the structural evaluation. Moreover, it can influenced the absence of affinity of the coating for the steam sterilization and/or hydrogen peroxide action, as identified in this study.

## 4. Discussion

Acrylic polymers are a highly varied category of synthetic substances. Since the initiation of industrial production in 1927, the polymerization of freshly synthesized monomers has facilitated the acquisition of materials with notable features and applications. Acrylic polymer-based products are utilized in the manufacturing of packaging for food, textiles, electronics, automotive applications, and pharmaceuticals. Nonetheless, such extensive use generates a substantial quantity of trash, which is frequently challenging to handle. Acrylic polymers are utilized in the coating of textiles with acrylic resins. The use of coatings on textiles, especially fabrics, alters their qualities, such as enhancing color fastness and improving resistance to environmental influences. The extensive utilization of the aforementioned fabrics produces significant quantities of post-production waste. Applying synthetic compounds to fabrics enhances waste resistance to degradation and inhibits the recycling of material waste. The extraction of polyacrylic resin from textile surfaces can facilitate material reuse, hence diminishing waste, which is critically significant from an ecological perspective. The findings indicate that biotechnological approaches are effective for the removal of acrylic coatings from waste fabrics. The *Gordonia alkanivorans S7* strain, which originated from the Institute of Molecular and Industrial Biotechnology (TUL), facilitates the degradation of resin layers on material surfaces, achieving high yields in coating removal without the need for pre-chemical treatment of samples. This novel approach offers a sustainable alternative for the management and recycling of coated textile materials, tackling a major difficulty in textile waste processing. This method facilitates the recovery of fibers from coated fabrics, thereby supporting the circular economy and fostering environmentally sustainable waste management. A biotechnological method for removing resin layers from used materials can be developed, potentially reducing post-production waste.

The selection of suitable microorganisms and enzyme preparations, along with their application methods, can result in the complete mineralization of resins. The utilized strain is recognized for its degradative activity on petroleum compounds, suggesting that the residual biomass post-process may be applicable in bioremediation efforts, indicating that the method developed by this project has potential for waste-free outcomes.

Both qualitative and quantitative studies have demonstrated the effectiveness of the acrylic resin removal process from woven fabrics classified as post-production or post-use waste. The ATR-FTIR study confirmed the reduction in the mass of the tested fabrics, indicating the absence of the absorbance band associated with acrylic coatings in biodegraded textiles. The biotechnological process developed for the removal of acrylic coatings shows potential for recovering initial PET fibers from manufacturers and mitigating their environmental impact.

## 5. Conclusions

The presented studies show the possibility of using a biotechnological method via the *Gordonia alkanivorans* S7 strain for the efficient removal of acrylic resin coatings from textile waste. The developed method enables fiber recovery, which contributes to sustainable waste management. The evolved biotechnological process eliminates polymer layers from the surface of the material, even without chemical pretreatment, which is confirmed via ATR-FTIR spectroscopy (Thermo Fisher Scientific, Waltham, MA, USA). This approach makes the process an environmentally friendly alternative to conventional recycling methods. Moreover, due to the broad degradation activity of the strain used, it is possible to use the residual biomass as a preparation for bioremediation, creating the possibility of developing a fully circular and waste-free textile recycling strategy. Future studies should focus on optimizing the process parameters in order to increase biodegradation efficiency and assess its industrial feasibility on a large scale. 

## Figures and Tables

**Table 1:** Protein concentration in the culture medium during the biodegradation of coated textile fabrics.

Sample	Protein Concentration [µg/cm^3^]		
	Biodegradation Time [h]		
	24	48	72
SB	0.450 ± 0.013	0.520 ± 0.016	0.720 ± 0.002
SHPB	0.260 ± 0.008	0.320 ± 0.009	0.430 ± 0.013
SHP	0.012 ^(1)^	0.021 ^(1)^	0.021 ^(1)^
S	0.015 ^(1)^	0.018 ^(1)^	0.020 ^(1)^

^(1)^ the protein was not detected. SB—the material was sterilized and then biodegraded using the tested strain of bacteria; SHPB—the material was sterilized, treated for 30 min in 6% H_2_O_2_, washed with sterile distilled water, and then biodegraded; SHP—the material was sterilized and then treated only for 30 min in 6% H_2_O_2_; S—the material was sterilized. The mass reduction for samples 3 and 4 was not observed in all cases of the treatment.

**Table 2:** Mass loss of the coated polyester textiles.

Treatment Variant	Mass Reduction After Treatment [wt%]
SB	6.90 ± 0.02
SHPB	5.81 ± 0.02
SHP	0.0
S	0.0

SB—the material was sterilized and then biodegraded using the tested strain of bacteria; SHPB—the material was sterilized, treated for 30 min in 6% H_2_O_2_, washed with sterile distilled water, and then biodegraded; SHP—the material was sterilized and then treated only for 30 min in 6% H_2_O_2_; S—the material was sterilized. The mass reduction for samples 3 and 4 was not observed in all cases of the treatment.

**Table 3:** The characteristic absorption bands identified in the ATR-FTIR spectra of the coated woven fabrics before biodegradation (control sample C). SB—the coated textile fabric was sterilized and then biodegraded using the tested strain of bacteria; SHPB—the material was sterilized, treated for 30 min in 6% H_2_O_2_, washed with sterile distilled water, and then biodegraded.

Fuctional Group	C (Initial Coated Woven Fabric)	SB	SHPB
	Wavenumber [cm^−1^]		
-OH of carobxyl group	3350	3390	3233
-CH_2_	2957, 2931	2958, 2959	2959, 2909
C=O carboxyl from ester group	1726	1713	1713
C=C benzene ring	1579, 722	1578, 722	1550, 722
Aromatic ring	1379	1408	1408
C-O	1241	1242	1242
C-O	1158	-	-
C-O-C	1098	1095	1096
C-OH	1018	1017	1017
Five hydrogen atoms substituted in an aromatic ring	871, 845	871, 846	871, 847

## Data Availability

The raw data supporting the conclusions of this article will be made available by the authors upon request due to privacy/ethical reasons.
